# Supplementary material for: Superior pedal function recovery of newly designed three spike insole over total contact insole in refractory plantar fasciitis: A randomized, double-blinded, non-inferiority study
Source: PLoS One. 2021 Jul 23;16(7):e0255064. doi: 10.1371/journal.pone.0255064 (PMC8301654; doi:10.1371/journal.pone.0255064)
Supplement: S1 File — (DOC) [file pone.0255064.s002.doc]

| **임상연구 계획서** |
| --- |
| **(1)임상시험의 명칭 및 단계** |
| 국문: 난치성 족저 근막염 환자에서 3D 프린팅 기술을 이용한 일반 전접촉 안창과 족저 근막염 안창의 효과 비교; 전향적, 무작위적 대조 연구  영문: Comparative study of effectiveness between typical total contact insole and plantar fasciitis specific insole using 3D printing technology; Prospective and randomized study |
| **(2)임상시험 실시기관명 및 주소** |
| 실시기관명: 가톨릭관동대학교 국제성모병원  주소: 인천시 서구 심곡로 100번길 25 국제성모병원 |
| **(3)임상시험의 책임자 및 담당자/공동연구자 성명, 직위, 소속** |
| | **구분** | **성명** | **소속** | **직위** | **연락처(휴대폰)** | | --- | --- | --- | --- | --- | | 연구책임자 | 심동우 | 가톨릭관동대학교 정형외과 | 임상조교수 | 010-7110-7430 | | 코디네이터 | 강경일 | 가톨릭관동대학교  세포치료센터 | 간호사 | 010-2378-6597 | |
| **(4)임상시험용 의약품 등을 관리하는 약사의 성명 및 직위, 소속** |
| 해당사항 없음 |
| **(5)의뢰자(임상시험을 하려는 자, 기관)의 명칭 및 주소** |
| 의뢰자: 가톨릭관동대학교 정형외과 심동우  주소: 인천시 서구 심곡로 100번길 25 국제성모병원 |
| **(6) 임상시험의 목적** |
| 족저근막염 환자에 대해 통상적인 인솔 치료 환자와 3D프린팅 기술을 활용 Plantar fascia-specific stretch이 가능한 환자 맞춤형 Insole 착용환자의 visual analog scale, Roles and Maudsley score, American Orthopaedic Foot and Ankle Society (AOFAS) score, SF 36 Global health Rating Scale 등을 측정하여 두 군간의 치료 효과를 비교함으로써 족저근막염에서 새로운 디자인의 Insole의 유효성을 검증하고자 한다. |
| **(7) 임상시험의 배경** |
| 족저 근막염은 흔한 족부 질환의 하나로 만성적이며, 기능상 장애를 초래할 수 있으나 정확한 병리 기전은 밝혀진 바 없으며 이에 관하여 근위 근막 두께의 증가 및 혈류의 감소, 건 주위 염증 소견 및 통증 수용체의 변화 등이 환자의 증상을 유발하는 요소로 추정된다. 이 외에도 외상, 적절치 못한 신발의 착용 및 비만, 장시간 서서 일하는 직업 등이 영향을 미칠 것으로 생각된다. 그 원인으로는 근위부에서 족저 근막이 자극을 많이 받아 발생하게 되는데 우선 과체중의 환자에서 후족부가 회내전 상태로 되면서 족저 근막이 종지하는 부위에 자극을 받아서 발생하는 경우와 또 다른 기전으로는 체중 부하는 많지 않지만 근막 다발이 경직되면서 족저 근막에 자극이 많이 가는 유형으로 이 때는 걷거나 뛸 때 족근관절의 배측굴곡 대신 중족부나 전족부가 배측굴곡 되면서 족저 근막이 당겨져 종지하는 종골 내측부위의 염증성 반응을 일으키는 것으로 설명되고 있다. 그 외에도 내측 종골 신경의 자극 및 포착이나 후족부 내측부의 인대 및 신경 부위가 미세 외상에 반복적으로 노출이 되면서 통증이 발생한다는 주장도 있다. 특징적으로 환자가 아침에 첫 발을 디딜 때 통증이 가장 심하고 낮 동안 증상이 지속되거나 호전되며 일상 생활을 하면서 더 악화되기도 한다. 방사선학적 검사상 전체의 약 50% 정도에서 석회화 및 종골의 골극(bony spur)을 보이게 되나 증상이 없는 환자의 약 20% 정도에서도 같은 변형을 보일 수 있으므로 진단적 가치는 낮다. 중년의 남, 여에 같은 비율로 발생하는 것으로 알려져 있고 약 10%에서는 양측에 발생하며, 80% 이상에서 보존적 치료만으로 1년 이내에 호전이 된다. 이러한 경과를 볼 때대 부분에서 보존적 치료로 호전되는데 스트레칭, 얼음찜질, 보조기, 스테로이드 주사 등을 사용할 수 있다. 이러한 보존적 치료에 6주 이상 효과가 없을 시 체외 충격파 요법, 수술적 치료를 고려해 볼 수 있다. 그 결과에 대해서는 저자들마다 득과 실에 대하여 다양한 보고들이 있다.  환자 맞춤형 3D 프린팅 기술을 최근에 의료에서 각광받고 있으며, 이 기술을 활용하여, 족저 근막염 환자가 일상생활을 하면서, 통증조절에 효과가 있는 스트레칭을 효율적으로 하기 위해, 족저근막염 환자의 스트레칭 치료를 위한 환자 맞춤형 안창을 제작 및 적용하여 치료결과의 차이를 보고 하고자 한다. |
| **(8) 임상시험용 의약품등의 코드명이나 주성분의 일반명, 원료약품 및 그 분량, 제형 등** |
| 해당사항없음. |
| **(9) 연구 대상 및 대상 질환** |
| 족저 근막염으로 통증을 호소하며, 보존적 치료를 시행중인 자 |
| **(10) 대상자의 선정기준, 제외기준, 목표한 대상자의 수 및 그 근거** |
| - 선정기준  1. 6주 이상의 보존적 치료(스트레칭, 휴식, 냉찜질, 진통제, 보조기)에 효과가 없는 19세 이상 성인 환자 2. 아침에 일어나서 처음 걷는 몇 분간의 Visual Analog Scale (VAS)이 5이상 3. Baseline Roles and Maudsley Score of 3 or 4 4. 대상자 동의서에 서명한 환자  - 제외기준  1. 1개월 이내에 스테로이드 주사를 맞은 경우 2. 6주 이상의 보존적 치료 및 스테로이드 주사, 맞춤형 안창, 야간부목 치료에도 호전이 없고, 6개월이상 지속되어 수술적 치료가 필요한자 3. 이전에 해당 부위에 감염의 과거력이 있거나 3개월 이내 해당부위에 시술의 과거력이 있는 경우 4. 후경골 동맥과 족배 동맥의 맥박이 촉지 되지 않는 경우 5. 강직성 척추염 및 류마티스 관절염 등의 전신성 염증질환, 신경 포착 증후군, 아킬레스건염, 출혈성 경향, 거골하 관절염 및 족부 변형에 의한 후족부 통증을 가진 환자 및 피부 병변이나 상처가 있는 경우 6. 종골의 피로 골절이 있는 경우 7. 문맹, 외국인 등 동의서를 읽을 수 없는 경우 8. 연구 참여를 거부한자 9. 기존의 피부질환이 있는자  - 목표한 대상자의 수 및 그 근거   대상자의 수: 총 24명의 환자를 두 집단으로 나누어 분류한다. 그룹1: 통상적인 전접촉 안창 적용군 (대조군 n=(12) ) 그룹2: 족저근막염 특화 안창 적용군 (실험군 n=(12) )  산출 근거: 전접촉 안창을 사용한 군과 편평 안창을 사용한 군을 비교한 기존 연구에서 치료 6개월 후 Visual Analog Scale (VAS)의 평균 차이는 1.56였으며 표준편차는 1.31 이었다. 본 연구에서 통상적인 전접촉 안창을 사용한 군과 족저 근막염 특화 안창을 사용한 군의 VAS 차이가 1.6 차이가 날 것으로 가정할 때, α는 0.05, power는 80%일 경우, PASS software (power analysis and sample-e package, NCSS statistical software)를 이용하여 표본수를 구하면 군당 11명이 요구된다. 중도 탈락률을 10% 고려하여 각 군당 12명으로 연구를 진행한다. 따라서 총 24명으로 연구를 진행하고자 한다. |
| **(11) 임상시험의 기간** |
| IRB 승인 후 ~ 1년 |
| **(12)임상시험의 방법(투여·사용량, 투여·사용 방법, 투여·사용 기간, 병용요법 등)** |
| - 대조군  1. 족저근막염의 환자 중 선정/제외기준에 적합한 대상자에게 연구 참여 동의서를 획득한다. 2. 컴퓨터를 이용한 무작위 추출법으로 실험군과 대조군을 나눈다. 3. 체중이 실린 환자의 족부 표면을 안창 제작용 틀을 이용하여 획득한다. 4. 3D 모델링 기술을 이용하며 족부 표면에 대한 3D 모델링 Data를 수집한다. 5. 3D 모델링 Data를 이용하여, 통상적인 전접촉 안창을 제작한다. 6. 제작 된 환자 맞춤형 안창을 적용 후 6주, 3개월, 6개월, 1년에 걸쳐 visual analog scale(VAS), modified Roles and Maudsley score, American Orthopaedic Foot and Ankle Society (AOFAS) ankle-hindfoot score, SF-36 scale 측정한다.  - 실험군  1. 족저근막염의 환자 중 선정/제외기준에 적합한 대상자에게 연구 참여 동의서를 획득한다. 2. 컴퓨터를 이용한 무작위 추출법으로 실험군과 대조군을 나눈다. 3. 체중이 실린 환자의 족부 표면을 안창 제작용 틀을 이용하여 획득한다. 4. 3D 모델링 기술을 이용하며 족부 표면에 대한 3D 모델링 Data를 수집한다. 5. 3D 모델링 Data를 이용하여, 족저 근막염 특화 안창을 제작한다. 6. 제작 된 지압판이 달린 환자 맞춤형 안창을 적용 후 6주, 3개월, 6개월, 1년에 걸쳐 visual analog scale(VAS), modified Roles and Maudsley score, American Orthopaedic Foot and Ankle Society (AOFAS) ankle-hindfoot score, SF-36 scale 측정한다. |
| **(13) 관찰항목, 임상검사항목 및 관찰 검사 방법** |
| - 임상적 계측은 통상적인 보존적 치료군과 환자 맞춤형 안창 착용군을 6주, 3개월, 6개월, 1년에 걸쳐 조사를 실시한다.  - 항목은 각각 visual analog scale(VAS), modified Roles and Maudsley score, American Orthopaedic Foot and Ankle Society (AOFAS) ankle-hindfoot score, SF-36 scale을 측정한다. |
| **(14) 예측 부작용 및 사용상의 주의사항** |
| 족저근막염 환자의 인솔은 환자 본인이 임의로 착용을 하는 것이 일반적으로 비침습적인 방법이다. 3D 프리팅 재질은 인체에 적합한 물질(TPU)로 예측되는 부작용은 없다. 단, 기존의 피부질환자는 제작되는 안창 재질에 대한 피부질환 악화 또는 예측하지 못한 부작용이 나타날 수 있어 대상자에서 제외한다. |
| **(15) 대상자의 임상시험 참여 중지 및 탈락 기준** |
| 환자의 자발적인 탈락 의사에 따라 임상시험 중지를 결정한다. |
| **(16) 효과 평가기준, 평가방법, 해석방법(통계분석방법)** |
| - 효과 평가기준   족저 근막염의 줄기세포 치료전후 임상적 평가를 시행한다.   - 평가방법   맞춤형 안창 착용 전 visual analog scale, Roles and Maudsley score, American Orthopaedic Foot and Ankle Society (AOFAS) score, SF 36 Global health Rating Scale을 기록하며, 착용 후 6주, 3개월, 6개월, 12개월 단위로 상기 계측을 기록한다.   - 통계분석방법   안창 착용 전과 추적관찰 기간의 임상적 계측의 비교는 SPSS 프로그램 ver. 21.0 (SPSS Inc., Chicago, IL, USA)의 그룹간 Wilcoxon signed rank test, 그룹내 Mann Whitney test 를 이용하여 분석한다. |
| **(17) 부작용을 포함한 안전성의 평가기준, 평가방법 및 보고방법** |
|  |
| **(18) 임상시험 종료 후 대상자의 진료 및 치료기준** |
| - 일반적인 족저 근막염 환자의 추시 관찰 과정을 따른다. - 기존에 정해진 추시 기간에 환자를 진찰하고 검사를 진행한다. |
| **(19) 피해자 보상에 대한 규약** |
| 환자 맞춤형 안창 대한 제작 비용은 일체 연구자가 부담하여, 불편감 및 자의로 인한 탈락시 임상시험을 종료하며, 이에 대한 피해자 보상은 없다. |
| **(20) 대상자의 안전보호에 관한 대책** |
| 해당 연구에 참여하는 기간 동안 대상자에게 생기는 부작용은 즉각 연구책임자에게 연락한다. 그러나 주사 치료는 외래에서 족저근막염이나 만성 건염이 있는 환자에게 안전하게 이용되고 있으며 부작용의 발생 가능성이 낮고, 부작용이 발생 하더라도 쉽게 해결할 수 있는 범주의 문제로 연구의 위험수위가 level II 이기 때문에 별도의 독립적인 위험감시위원회는 필요하지 않을 것으로 생각된다. 단, 유해사례 발생시 피험자가 임상시험 처치로 인한 이상반응 발생여부, 증상, 발현일, 소실일, 정도, 임상시험용의료기기와의 관련성, 관련된 조치, 치료, 결과 등 확인된 사항들을 증례기록서(case report form)에 기재한다. 이상반응의 확인 및 보고는 임상시험에 참가하고 있는 모든 피험자의 이상반응은 매 방문 때 마다 발생 여부를 확인하고, 이를 발견하였을 때는 의무기록지와 증례기록서의 해당란에 상세히(시작일, 종료일, 중증도, 임상시험용의료기기와의 관련성, 치료여부, 결과) 기재되어야 한다. 임상시험기간 동안에 발생한 모든 이상반응은 임상시험 완료일까지 추적 조사 한다. |
| **(21) 그 밖에 임상시험을 안전하고 과학적으로 실시하기 위하여 필요한 사항** |
| - 공동 연구자 및 참여자의 반복적인 교육 - 예측하지 못한 합병증에 대한 비참여 전문가의 자문 |
| **(22) 해당연구의 근거가 되는 임상문헌(참고 문헌)** |
| 1. Fong, DT, Pang, KY, Chung, MM, Hung, AS, Chan, KM. Evaluation of combined prescription of rocker sole shoes and custom-made foot orthoses for the treatment of plantar fasciitis. *Clin Biomech (Bristol, Avon)*. 2012;27(10):1072-1077.  2. Landorf, KB, Keenan, AM, Herbert, RD. Effectiveness of foot orthoses to treat plantar fasciitis: a randomized trial. *Arch Intern Med*. 2006;166(12):1305-1310.  3. Oliveira, HA, Jones, A, Moreira, E, Jennings, F, Natour, J. Effectiveness of total contact insoles in patients with plantar fasciitis. *J Rheumatol*. 2015;42(5):870-878.  4. Roos, E, Engstrom, M, Soderberg, B. Foot orthoses for the treatment of plantar fasciitis. *Foot Ankle Int*. 2006;27(8):606-611.  5. Wrobel, JS, Fleischer, AE, Crews, RT, Jarrett, B, Najafi, B. A randomized controlled trial of custom foot orthoses for the treatment of plantar heel pain. *J Am Podiatr Med Assoc*. 2015;105(4):281-294.  6. Yucel, U, Kucuksen, S, Cingoz, HT, et al. Full-length silicone insoles versus ultrasound-guided corticosteroid injection in the management of plantar fasciitis: a randomized clinical trial. *Prosthet Orthot Int*. 2013;37(6):471-476. |

- 해당사항이 없는 항목은 ‘해당사항 없음’으로 기재해 주십시오.
- 임상시험/연구와 관련하여 보충 설명/자료가 필요한 경우 별첨하여 주시기 바랍니다.
