# Supplementary material for: Superior pedal function recovery of newly designed three spike insole over total contact insole in refractory plantar fasciitis: A randomized, double-blinded, non-inferiority study
Source: PLoS One. 2021 Jul 23;16(7):e0255064. doi: 10.1371/journal.pone.0255064 (PMC8301654; doi:10.1371/journal.pone.0255064)
Supplement: S2 File — (DOC) [file pone.0255064.s003.doc]

| **Study Protocol** |
| --- |
| **(1)Title** |
| 국문: 난치성 족저 근막염 환자에서 3D 프린팅 기술을 이용한 일반 전접촉 안창과 족저 근막염 안창의 효과 비교; 전향적, 무작위적 대조 연구  영문: Comparative study of effectiveness between typical total contact insole and plantar fasciitis specific insole using 3D printing technology; Prospective and randomized study |
| **(2)Institution & addresss** |
| Institution: Catholic Kwandong University International St.Mary’s Hospital  Address: 25, Simgok-ro 100beon-gil, Seo-gu, Incheon, 22711, South Korea |
| **(3) Name, position, and affiliation of the person in charge of clinical trials and the person in charge/co-researcher** |
| | **Role** | **Name** | **Department** | **Title** | **Phone** | | --- | --- | --- | --- | --- | | Chief of Research | Dong Woo Shim | Orthopaedic Surgery | Assistant Professor | 010-7110-7430 | | Coordinator | Kyung-Yil Kang | Department of Cell Therapy | Registered nurse | 010-2378-6597 | |
| **(4)** **Name, position, and affiliation of a pharmacist who manages medicines, etc. for clinical trials** |
| N/A |
| **(5) Name and address of the conductant** |
| conductant: Dong Woo Shim  Address: 25, Simgok-ro 100beon-gil, Seo-gu, Incheon, 22711, South Korea |
| **(6) Purpose of the study** |
| It is intended to verify the validity of patient-tailored insoles in plantar fasciitis by comparing the treatment effects between the two groups by measuring the clinical measurement of patient-tailored insoles using 3D printing technology and conventional insole. |
| **(7) Background** |
| Plantar fasciitis is a common pedigree disease that can lead to dysfunction, but no exact pathology has been identified, and it is assumed that increased proximal fascia thickness, decreased blood flow, and changes in peripheral inflammatory findings and pain receptors cause symptoms. In addition, trauma, inappropriate wearing and obesity of shoes, and long standing jobs are expected to affect it. The reason for this is that the proximal fascia is highly stimulated in overweight patients, and other mechanisms are not much weight load, but when walking or running, the fascia is pulled. In addition, some argue that the irritation of the inner tibial nerve and the pain occurs due to repeated exposure to microtrauma in the capture or ligaments and nerves in the inner part of the posterior part. Characteristically, when a patient takes his first step in the morning, the pain is the most severe, the symptoms persist or improve during the day, and sometimes get worse in daily life. Radiological tests show bony spurs in about 50% of the total, but the diagnostic value is low because about 20% of patients with no symptoms may exhibit the same deformation. It is known to occur in the same proportion as in middle-aged men and women, and in about 10% it occurs on both sides, and in more than 80% it is improved within a year with only conservative treatment. Most of these developments improve with conservative treatments, such as stretching, ice packs, aids, and steroid injections. In vitro shock wave therapy and surgical treatment can be considered if these preservation treatments are ineffective for more than six weeks. Each author has various reports of gain and loss on the results. Patient-tailored 3D printing technology has recently been spotlighted in healthcare, and we want to use this technology to report differences in treatment results by creating and applying patient-tailored insole for stretching plantar fasciitis to efficiently stretch in their daily lives. |
| **(8) Code name, general name of the main ingredient, raw material, amount of the drug, formulation, etc. for clinical trials;** |
| N/A |
| **(9) Subject to study and target diseases** |
| A person who complained of pain and is undergoing conservative treatment due to plantar fasciitis. |
| **(10) Selection criteria, exclusion criteria, number of target persons and grounds for** |
| - Inclusion criteria  1. Adult patients aged 19 or older who are not effective in preserving treatment (stretching, resting, cold steaming, painkillers, aids) for at least 6 weeks 2. The Visual Analog Scale (VAS) of the first few minutes after waking up in the morning is 5 or higher. 3. Baseline Roles and Maudsley Score of 3 or 4 4. Patients who have signed the consent form for the subject  - Exclusion criteria  1. In case of steroid injection within one month 2. A person who needs surgical treatment for more than six weeks of preservation treatment, steroid injections, customized eyeglasses, and nighttime splint treatment, which is not good for more than six weeks and lasts for more than six months. 3. Previously, if the area has a history of infection or if the area has a history of treatment within three months 4. If the pulse of the posterior tibial artery and dorsalis pedis artery is not accelerated. 5. Patients with systemic inflammatory diseases, such as ankylosing spondylitis and rheumatoid arthritis, nerve entrapment syndrome, Achilles tendinitis, hemorrhagic tendencies, subtalar arthritis, or hindfoot deformity, or skin lesions or wounds; 6. Stress fracture of calcaneus 7. Where it is impossible to read the consent form, such as illiteracy, foreigners, etc. 8. Those who refused to participate in the study 9. Persons with existing skin diseases  - Number of target and their rationale   Number of subjects: A total of 24 patients are divided into two groups.  Group1: Typical total contact insole (control group n=12)  Group 2: Plantar fascial specific insole (experimental group n=12)  Rationale: In a previous study comparing groups using total contact insole and groups using flat insole, the average difference in Visual Analog Scale (VAS) after six months of treatment was 1.56 and the standard deviation was 1.31. In this study, assuming 1.6 difference of VAS between typical total contact insole and plantar fascial specific insole, when α = 0.05, power is 80%, a sample count required by using PASS software (power analysis and sample-e package, NCSS statistical software) was 11 patients in each group. The research will be conducted with 12 people per gruop, considering the dropout rate of 10%. Therefore, we would like to conduct the study with a total of 24 people. |
| **(11) Period of clinical trials** |
| After IRB approval ~ 1year |
| **(12) Methods** |
| - Control  1. Consent to participate in the study is obtained from patients with plantar fasciitis who meet the criteria for selection/exclusion 2. Using computerized randomization method, divide the experimental group and control group. 3. The pedigree surface of a patient with weight is obtained using a frame for insole production. 4. 3D modeling technology is utilized and 3D modeling data is collected for the pedigree surface. 5. Using 3D modeling data, make a typical total contact insole. 6. Measure visual analog scale(VAS), modified Roles and Maudsley score, American Orthopaedic Foot and Ankle Society (AOFAS) ankle-hindfoot score, SF-36 scale over a period of six weeks, three months, six months, and one year after application.  - Experimental group   1) Consent to participate in the study is obtained from patients with plantar fasciitis who meet the criteria for selection/exclusion  2)Using computerized randomization method, divide the experimental group and control group.  3)The pedigree surface of a patient with weight is obtained using a frame for insole production.  4) 3D modeling technology is utilized and 3D modeling data is collected for the pedigree surface.  5) Using 3D modeling data, make a newly designed insole.   1. Measure visual analog scale(VAS), modified Roles and Maudsley score, American Orthopaedic Foot and Ankle Society (AOFAS) ankle-hindfoot score, SF-36 scale over a period of six weeks, three months, six months, and one year after application. |
| **(13) Observation items, clinical test items, and observation test methods** |
| - Clinical outcome conducts surveys over six weeks, three months, six months, and one year for the usual preservation treatment group and the patient-tailored insole group.  - The items are each visual analog scale(VAS), modified Roles and Maudsley score, American Orthopaedic Foot and Ankle Society (AOFAS) ankle-hindfoot score, SF-36 scale |
| **(14) Predictive side effects and precautions for use** |
| Insole in patients with plantar fasciitis is generally a non-invasive method for the patient to wear it arbitrarily. 3D printing material has no side effects that are expected to be suitable for human body (TPU). However, existing skin problem patients are excluded from the subjects because of the deterioration of the skin disease or unforeseen side effects on the fabricated ophthalmic material. |
| **(15) Criteria for suspension and elimination of participation in clinical trials by subjects** |
| The patient's willingness to voluntarily drop out determines the suspension of clinical trials. |
| **(16) Statistical analyses** |
| Statistical analyses  The comparison of clinical outcome before wearing the insole and during the follow-up period is analyzed using the Wilcoxon signed Rank test and the Mann Whitney test using SPSS program ver.21.0 (SPSS Inc., Chicago, IL, USA), and the group. |
| **(17) Standards, evaluation methods and reporting methods for safety including side effects** |
| Follow general IRB regulations |
| **(18) Medical treatment and treatment standards for the subject after the clinical trial is completed** |
| Follow the follow-up observation process of a general plantar fasciitis patient.  Patients are examined and examined during the previously defined follow-up period. |
| **(19) Conventions for compensation for victims.** |
| The researcher pays for the production of patient-tailored insole, and the clinical trial will be terminated in case of inconvenience or voluntary elimination, and there is no compensation for the victim. |
| **(20) Measures for the safety protection of persons subject to the examination** |
| Any side effects that occur to the subject during the period of participation in the study are immediately contacted by the research manager. However, insole therapy is not considered dangerous because it is safe for patients with outpatient to plantar fasciitis or chronic tendinitis, and the risk level of the study is level II due to the low probability of adverse events. However, in the case report, the identified matters such as whether or not an abnormal reaction occurs due to clinical trial treatment, symptoms, expression date, disappearance date, degree, relevance to clinical trial medical devices, related measures, treatment, and results are recorded. Verification and reporting of adverse events shall be conducted at each visit, and when found, detailed (start date, end date, severity, association with clinical trial medical devices, treatment status, results) shall be recorded in the corresponding column of the medical record. All adverse events during the clinical trial period shall be tracked and investigated until the completion of the clinical trial. |
| **(21) Other matters necessary for the safe and scientific conduct of clinical trials** |
| Repeated training of co-researchers and participants  Consultation by non-participating experts on unforeseen complications |
| **(22) References** |
| 1. Fong, DT, Pang, KY, Chung, MM, Hung, AS, Chan, KM. Evaluation of combined prescription of rocker sole shoes and custom-made foot orthoses for the treatment of plantar fasciitis. *Clin Biomech (Bristol, Avon)*. 2012;27(10):1072-1077.  2. Landorf, KB, Keenan, AM, Herbert, RD. Effectiveness of foot orthoses to treat plantar fasciitis: a randomized trial. *Arch Intern Med*. 2006;166(12):1305-1310.  3. Oliveira, HA, Jones, A, Moreira, E, Jennings, F, Natour, J. Effectiveness of total contact insoles in patients with plantar fasciitis. *J Rheumatol*. 2015;42(5):870-878.  4. Roos, E, Engstrom, M, Soderberg, B. Foot orthoses for the treatment of plantar fasciitis. *Foot Ankle Int*. 2006;27(8):606-611.  5. Wrobel, JS, Fleischer, AE, Crews, RT, Jarrett, B, Najafi, B. A randomized controlled trial of custom foot orthoses for the treatment of plantar heel pain. *J Am Podiatr Med Assoc*. 2015;105(4):281-294.  6. Yucel, U, Kucuksen, S, Cingoz, HT, et al. Full-length silicone insoles versus ultrasound-guided corticosteroid injection in the management of plantar fasciitis: a randomized clinical trial. *Prosthet Orthot Int*. 2013;37(6):471-476. |
