# Supplementary material for: Superior pedal function recovery of newly designed three spike insole over total contact insole in refractory plantar fasciitis: A randomized, double-blinded, non-inferiority study
Source: PLoS One. 2021 Jul 23;16(7):e0255064. doi: 10.1371/journal.pone.0255064 (PMC8301654; doi:10.1371/journal.pone.0255064)
Supplement: Attachment — Submitted filename: PONE-D-21-05601_reviewer_Edited.pdf [file pone.0255064.s005.pdf]

PLOS ONE

Superior pedal function recovery of newly designed three spike insole over total contact insole in refractory plantar fasciitis; a randomized, double-blinded, non-inferiority study

--Manuscript Draft--

**Manuscript Number:**	PONE-D-21-05601
**Article Type:**	Clinical Trial
**Full Title:**	Superior pedal function recovery of newly designed three spike insole over total contact insole in refractory plantar fasciitis; a randomized, double-blinded, non-inferiority study
**Short Title:**	Superior pedal function recovery of newly designed TSI over TCI in refractory plantar fasciitis
**Corresponding Author:**	Dong Woo Shim<br>Catholic Kwandong University International Saint Mary's Hospital<br>Incheon, KOREA, REPUBLIC OF
**Keywords:**	chronic plantar fasciitis, foot orthosis, conservative therapy, rehabilitation
**Abstract:**	Introduction: Plantar fasciitis is one of the common foot complaints that is chronic and can induce dysfunction. Total contact insole (TCI) is simple but effective in treating plantar fasciitis. Nevertheless of the effect, the cost and the long duration for production have been the major flaws. Therefore, we developed newly designed three-spike insole (TSI) that can be commercially productive and compared the clinical outcomes with TCI.<br>Methods: Patients with plantar fasciitis refractory to conservative treatment for more than 6 weeks were candidates. We produced insoles with hardness of 58 ± 5 Shore-A. Twenty-eight patients were randomly allocated to use either TSI or TCI evenly. The following assessment tools were used: visual analog scale (VAS), American Orthopaedic Foot and Ankle score, Foot and Ankle Outcome Score, Karlsson-Peterson (KP) score, Short Form-36 for quality of life, and Foot Function Index. Non-inferiority was declared if VAS was within the statistical variability of minimal important difference. A blinded assessor evaluated the groups at baseline and after 6, 12, and 24 weeks.<br>Results: The groups were homogenous for majority of variables at baseline. Overall patient-reported satisfaction showed improvement from mean 5.2 (range, 1 – 12) weeks of wearing and all clinical outcome scores showed significant improvements in both groups over time on the basis of Friedman test ($p < .05$). TSI showed non-inferiority to TCI at each time point. Post hoc analysis revealed that many scales showed significant superiority of TSI at 3 month ($p < .0125$) and KP score at 6 month ($p < .001$).<br>Conclusion: We reaffirmed that semi-rigid insole is effective in refractory plantar fasciitis and showed TSI restores pedal function more rapidly than TCI. TSI can be not only effective in deriving better clinical outcomes but also be manufactured for popularization to lower the price and producing time of orthosis.
**Order of Authors:**	Dong Woo Shim
	Seung-Yong Sung
	Whan-Yong Chung
	Kyung-Yil Kang
	Sung-Jun Park
	Jin woo Lee
	Dong Sik Chae
**Additional Information:**	
**Question**	**Response**
**Financial Disclosure**	DWS

Powered by Editorial Manager® and ProduXion Manager® from Aries Systems Corporation

Enter a financial disclosure statement that describes the sources of funding for the work included in this submission. Review the submission guidelines for detailed requirements. View published research articles from *PLOS ONE* for specific examples.<br><br>This statement is required for submission and **will appear in the published article** if the submission is accepted. Please make sure it is accurate.<br><br>**Unfunded studies**<br>Enter: *The author(s) received no specific funding for this work.*<br><br>**Funded studies**<br>Enter a statement with the following details:<br>• Initials of the authors who received each award<br>• Grant numbers awarded to each author<br>• The full name of each funder<br>• URL of each funder website<br>• Did the sponsors or funders play any role in the study design, data collection and analysis, decision to publish, or preparation of the manuscript?<br>• **NO** - Include this sentence at the end of your statement: *The funders had no role in study design, data collection and analysis, decision to publish, or preparation of the manuscript.*<br>• **YES** - Specify the role(s) played.<br><br>* typeset	This research was supported by Basic Science Research Program through the National Research Foundation of Korea(NRF) funded by the Ministry of Science, ICT & Future Planning(NRF-2019R1G1A1098885).<br><br>https://ernd.nrf.re.kr/index.do<br><br>The funders had no role in study design, data collection and analysis, decision to publish, or preparation of the manuscript.
**Competing Interests**<br><br>Use the instructions below to enter a competing interest statement for this submission. On behalf of all authors, disclose any competing interests that could be perceived to bias this work—acknowledging all financial support and any other relevant financial or non-financial competing interests.<br><br>This statement is required for submission and **will appear in the published article** if	The authors have declared that no competing interests exist.

the submission is accepted. Please make sure it is accurate and that any funding sources listed in your Funding Information later in the submission form are also declared in your Financial Disclosure statement.<br><br>View published research articles from *PLOS ONE* for specific examples.<br><br>**NO authors have competing interests**<br><br>Enter: *The authors have declared that no competing interests exist.*<br><br>**Authors with competing interests**<br><br>Enter competing interest details beginning with this statement:<br><br>*I have read the journal's policy and the authors of this manuscript have the following competing interests: [insert competing interests here]*<br><br>* typeset	
**Ethics Statement**<br><br>Enter an ethics statement for this submission. This statement is required if the study involved:<br><br>• Human participants<br>• Human specimens or tissue<br>• Vertebrate animals or cephalopods<br>• Vertebrate embryos or tissues<br>• Field research<br><br>Write "N/A" if the submission does not require an ethics statement.<br><br>General guidance is provided below. Consult the submission guidelines for detailed instructions. **Make sure that all information entered here is included in the Methods section of the manuscript.**	We have completed it. This study was approved by the Catholic Kwandong University 106International St. Mary's Institutional Review Board(IS18OISI0072)and registered in Clinical Research 107Information Service (KCT0004737, first registration (14/02/2020)).All methods were carried out in 108accordance with relevant guidelines and regulations and all experimental protocols were approved by a 109named institutional committee.

**Format for specific study types**<br><br>**Human Subject Research (involving human participants and/or tissue)**<br>• Give the name of the institutional review board or ethics committee that approved the study<br>• Include the approval number and/or a statement indicating approval of this research<br>• Indicate the form of consent obtained (written/oral) or the reason that consent was not obtained (e.g. the data were analyzed anonymously)<br><br>**Animal Research (involving vertebrate animals, embryos or tissues)**<br>• Provide the name of the Institutional Animal Care and Use Committee (IACUC) or other relevant ethics board that reviewed the study protocol, and indicate whether they approved this research or granted a formal waiver of ethical approval<br>• Include an approval number if one was obtained<br>• If the study involved *non-human primates*, add *additional details* about animal welfare and steps taken to ameliorate suffering<br>• If anesthesia, euthanasia, or any kind of animal sacrifice is part of the study, include briefly which substances and/or methods were applied<br><br>**Field Research**<br><br>Include the following details if this study involves the collection of plant, animal, or other materials from a natural setting:<br>• Field permit number<br>• Name of the institution or relevant body that granted permission	
**Data Availability**<br><br>Authors are required to make all data underlying the findings described fully available, without restriction, and from the time of publication. PLOS allows rare exceptions to address legal and ethical concerns. See the PLOS Data Policy and FAQ for detailed information.	No - some restrictions will apply

Powered by Editorial Manager® and ProduXion Manager® from Aries Systems Corporation

A Data Availability Statement describing where the data can be found is required at submission. Your answers to this question constitute the Data Availability Statement and **will be published in the article**, if accepted.<br><br>**Important:** Stating 'data available on request from the author' is not sufficient. If your data are only available upon request, select 'No' for the first question and explain your exceptional situation in the text box.<br><br>Do the authors confirm that all data underlying the findings described in their manuscript are fully available without restriction?	
**Describe where the data may be found in full sentences. If you are copying our sample text, replace any instances of XXX with the appropriate details.**<br><br>• If the data are **held or will be held in a public repository**, include URLs, accession numbers or DOIs. If this information will only be available after acceptance, indicate this by ticking the box below. For example: *All XXX files are available from the XXX database (accession number(s) XXX, XXX.)*.<br>• If the data are all contained **within the manuscript and/or Supporting Information files**, enter the following: *All relevant data are within the manuscript and its Supporting Information files.*<br>• If neither of these applies but you are able to provide **details of access elsewhere**, with or without limitations, please do so. For example:<br><br>*Data cannot be shared publicly because of [XXX]. Data are available from the XXX Institutional Data Access / Ethics Committee (contact via XXX) for researchers who meet the criteria for access to confidential data.*<br><br>*The data underlying the results presented in the study are available from (include the name of the third party*	The datasets generated and analyzed during the current study are not publicly available but are available from the corresponding author on reasonable request.

and contact information or URL).<br>• This text is appropriate if the data are owned by a third party and authors do not have permission to share the data.<br><br>* typeset	
Additional data availability information:	

Manuscript

Click here to access/download;Manuscript;Manuscript_PLOS one.docx

Superior pedal function recovery of newly designed three spike insole over total contact insole in refractory plantar fasciitis; a randomized, double-blinded, non-inferiority study

Short title: Superior pedal function recovery of newly designed TSI over TCI in refractory plantar fasciitis

Dong Woo Shim[1]

Seung-Yong Sung[1]

Whan-Yong Chung[1]

Kyung-Yil Kang[2]

Sung-Jun Park[3]

Jin Woo Lee[4]

Dong Sik Chae[1]

[1]Department of Orthopedic Surgery, International St. Mary's Hospital, Catholic Kwandong University College of Medicine (25, Simgok-ro 100beon-gil, Seo-gu, Incheon 22711, South Korea)

[2]Department of Medicine, Catholic Kwandong Graduate School (24, Beomil-ro 579beon-gil, Gangneung-si, Gangwon-do, 25601, South Korea)

[3]School of Mechanical, Automotive and Aeronautical Engineering Korea National University of

Transportation (50 Daehangno, Chungju, Chungbuk, 27469, South Korea)

[4]Department of Orthopaedic Surgery, Yonsei University College of Medicine (50 Yonsei-ro, Seodaemun-gu, Seoul, 03722, South Korea.)

Corresponding author

E-mail: drchaeos@gmail.com (DSC)

Author Contributions:

1. Conceptualization: Dong Woo Shim, Jin Woo Lee
2. Data curation: Kyung-Yil Kang, Dong Woo Shim
3. Formal analysis: Seung-Yong Sung, Whan-Yong Chung
4. Funding acquisition: Sung-Jun Park
5. Investigation: Dong Woo Shim, Whan-Yong Chung, Seung-Yong Sung
6. Methodology: Whan-Yong Chung, Jin Woo Lee
7. Project administration: Jin Woo Lee
8. Resources: Whan-Yong Chung, Jin Woo Lee
9. Software: Dong Woo Shim, Seung-Yong Sung
10. Supervision: Jin Woo Lee, Dong Sik Chae
11. Validation: Sung-Jun Park, Dong Sik Chae
12. Visualization: Dong Woo Shim, Whan-Yong Chung, Seung-Yong Sung
13. Writing – original draft: Dong Woo Shim
14. Writing – review & editing: Dong Sik Chae

¶These authors contributed equally to this work.

Abstract

Introduction: Plantar fasciitis is one of the common foot complaints that is chronic and can induce dysfunction. Total contact insole (TCI) is simple but effective in treating plantar fasciitis. Nevertheless of the effect, the cost and the long duration for production have been the major flaws. Therefore, we developed newly designed three-spike insole (TSI) that can be commercially productive and compared the clinical outcomes with TCI.

Methods: Patients with plantar fasciitis refractory to conservative treatment for more than 6 weeks were candidates. We produced insoles with hardness of 58 ± 5 Shore-A. Twenty-eight patients were randomly allocated to use either TSI or TCI evenly. The following assessment tools were used: visual analog scale (VAS), American Orthopaedic Foot and Ankle score, Foot and Ankle Outcome Score, Karlsson-Peterson (KP) score, Short Form-36 for quality of life, and Foot Function Index. Non-inferiority was declared if VAS was within the statistical variability of minimal important difference. A blinded assessor evaluated the groups at baseline and after 6, 12, and 24 weeks.

Results: The groups were homogenous for majority of variables at baseline. Overall patient-reported satisfaction showed improvement from mean 5.2 (range, 1 – 12) weeks of wearing and all clinical outcome scores showed significant improvements in both groups over time on the basis of Friedman test ($p < .05$). TSI showed non-inferiority to TCI at each time point. Post hoc analysis revealed that many scales showed significant superiority of TSI at 3 month ($p < .0125$) and KP score at 6 month ($p < .001$).

Conclusion: We reaffirmed that semi-rigid insole is effective in refractory plantar fasciitis and showed TSI restores pedal function more rapidly than TCI. TSI can be not only effective in deriving better clinical outcomes but also be manufactured for popularization to lower the price and producing time of orthosis.

Key words: *chronic plantar fasciitis, foot orthosis, conservative therapy, rehabilitation*

Introduction

Plantar fasciitis is one of the common foot complaints that is chronic and can induce dysfunction. Approximately 10% of population develops heel pain during their lifetime.[1] Distinguishing feature is the starting heel pain on the first step in the morning and it can wax and wane during the daytime. Approximately 90% of patients improve in a year with conservative management such as stretching exercise, night splint, orthotics, extracorporeal shockwave therapy, and local injection.[2-7] Recalcitrant plantar fasciitis, unresponsive to conservative care, can be treated with surgical intervention but the reports vary upon the outcomes.[8-11]

Foot orthosis is simple but effective in treating plantar fasciitis. It can be beneficial to patients in that population vulnerable to plantar fasciitis who tend to have a job with a long time walking or standing and tend to not have enough time spent on treatment including stretching exercise or visiting a clinician.[12] Many types of orthoses have been introduced but the efficacy among them are debatable. Some authors reported that prefabricated orthoses are ~~effective enough compared~~ to the customized foot orthoses.[13,14] However, majority of the studies showed superiority of the customized foot orthoses in the form of total contact insole to the prefabricated and sham orthoses.[15-20] Nevertheless of the effect, the cost and the long duration for production have been the major flaws. On average, it takes a week and costs 150 dollars for production.

Previous biomechanical studies have proposed that the medial surface of orthosis should stabilize the apical bony structure to effectively support the medial longitudinal arch and that the optimal insole contact surface should be over 54% of the plantar fascia to lessen the fascial stress and peak pressure.[21,22] Furthermore, structural analysis tells us that bending moments are higher in concentrated load model than in uniformly distributed load model.[23] Therefore, we developed newly designed insole that can be commercially productive, satisfying the above mentioned conditions. We hypothesized that the newly designed insole would not be inferior to the existing total contact insole and can have the merit to lower the

production price and time.

Methods

Study design

A parallel double-blinded randomized controlled study with a 6-month follow-up duration was carried out from February 2019 to April 2020. This study was approved by our institution. Prior to inclusion, all participants voluntarily participated in this study and gave written informed consent; and rights of participants were protected. Patients over 19-year-old with plantar fasciitis refractory to conservative treatment including stretching, resting, ice bag, and extracorporeal shock wave treatment (ESWT) etc. other than orthotics for more than 6 weeks were candidates for inclusion in this study. Furthermore, patients with visual analog scale (VAS) of 5 or higher pain when taking the first step were included. Exclusion criteria were previous insole use, injection around the foot and ankle within 3 months, pulselessness of dorsalis pedis and posterior tibial artery, other systemic inflammatory diseases, comorbidities that can mimic symptom such as Achilles tendinitis and subtalar arthritis, history of foot and ankle fracture, and hindfoot deformity. This study was registered on the clinical trials registry system and the manuscript was prepared according to the CONSORT statement. This study was approved by the Catholic Kwandong University International St. Mary's Institutional Review Board (IS18OISI0072) and registered in Clinical Research Information Service (KCT0004737, first registration (14/02/2020)). All methods were carried out in accordance with relevant guidelines and regulations and all experimental protocols were approved by a named institutional committee.

Twenty-eight patients were enrolled from the outpatient clinic and informed consents were obtained. An excel-generated block randomization list, which was created by a clinician not involved in this trial for concealment, was used to randomly allocate patients to the newly designed three-spike insole (TSI) group or total contact insole (TCI) group. A blinded physician assessed the outcome data.

Sample size

The sample size was calculated for a non-inferiority trial of 2 independent means between the groups using VAS as the main variable. For the determination of the minimal important difference (MID) of 2-cm on a 10-cm pain VAS, a 5% alpha set, 20% beta error, and standard deviation of 1.9 cm were established.[18,24] Twelve participants in each group were required for a minimal sample size; it was determined that each group consists of 14 patients, considering a possible loss of 10%.

Intervention

We used a 3-dimensional (3D) printer (Mandme, ®PLABS) to produce an insole. Both feet of all patients were copied with Copyfoam and the copies were sent to a manufacturer. All insoles were manufactured from thermoplastic polyurethane (TPU, density 12 g/cm^3) and had a 58 ± 5 Shore-A hardness filling up to 30% of the insole with TPU. Newly designed insole had three hemispheric spikes along the longitudinal medial arch starting from the center of plantar fascia. When a person's foot is 28.5 cm long, the measured length of plantar fascia in Copyfoam was about 12 cm. Therefore, each spike was designed to have a 2 cm diameter to support approximately 50% of the medial longitudinal arch; height of the first and third spike was 0.8 cm and that of the middle one was 1.2 cm. The first and the third spikes were placed at the most distal and proximal points of the medial longitudinal arch that were thought to be appropriate for stimulating the fascia on the basis of foot posture on Copyfoam. The distance between the spikes differed among the patients depending on the length of the patient's arch. Based on the initial copied foam, the total contact insole was manufactured to have structures supporting the arch on its medial border like the general customized total contact orthosis (Fig 1).

Fig 1. A, B) Three spike insole (TSI) and C, D) total contact insole (TCI)

At baseline, all participants were given either of the above-mentioned insoles according to the randomization for daily use for 6 months. They were instructed to wear it on shoes or slippers as much as they could in a day and were allowed to have no more intervention including stretching exercise during the research. All the participants were assessed by a blinded physician at baseline, 6 weeks, 3 months, and 6 months after wearing insole.

Clinical evaluation

Symptoms, pedal function, and foot and ankle related quality of life were the target evaluation. Clinical outcomes were assessed using maximal pain VAS in a day, American Orthopaedic Foot and Ankle Society (AOFAS) score, Foot and Ankle Outcome Score (FAOS), Karlsson-Peterson (KP) score, Medical Outcomes Study Short Form-36 (SF-36) for quality of life, and Foot Function Index (FFI).

Statistical Analysis

An intention to treat analysis was used — for the patients who were dropped out from further evaluations, the last data collected were used in the subsequent evaluations. Patient demographics and clinical outcomes are presented as mean ± SD or count (percentage). We used Mann-Whitney test for quantitative variables and Fisher's exact test for categorical variables for comparison between the groups. Non-inferiority test was analyzed to assess the VAS between 2 groups. Friedman test was used to compare between two groups over time. Post hoc analysis with Mann-Whitney test was conducted with a Bonferroni's correction, resulting in a significance level set at $p < .0125$. The level of statistical significance was set at $p < .05$. The Statistical Package for the Social Sciences (SPSS, version 21.0, IBM Corp., Armonk, NY, USA) was used for all statistical analyses.

Results

Twenty-eight patients were evenly distributed to 2 groups; a CONSORT flowchart is shown in Fig 2. Three participants were dropped from the study because of insole inconvenience and 1 participant was dropped for the treatment of radiating pain from herniated lumbar disc. Demographic characteristics are summarized in Table 1. The symptom duration and previous treatment duration tended to be longer in TSI group but were not significant. Mean age of all participants was 48.2 (range, 28 – 67), mean insole usage time was [illegible] (range, 2.5 – 12.0) hours a day, and mean time to clinical improvement was 5.2 (range, 1 – 12) weeks in all groups. Most common previous treatments were medication and ESWT. All participants took medicine for more than a month or underwent ESWT for more than 3 times; and 26 participants received both treatments. Three patients had previous local injection history around the plantar fascia for more than 3 months before enrollment.

Fig 2. CONSORT flowchart. Twenty-eight participants were distributed to 2 groups and 4 participants were lost to follow-up thoroughly. Twelve participants were evaluated in each group finally.

Table 1. Patient Demographics of newly designed TSI and TCI Groups

	TSI (N = 14)	TCI (N = 14)	P value
Age (years)	48.2 ± 9.9	48.2 ± 8.8	.999
Male gender	4 (28.6%)	4 (28.6%)	.999
Side (right: left: both)	5: 2: 7	6: 3: 5	.663
Height (cm)	162.9 ± 6.9	162.5 ± 8.2	.787
BMI (kg/m^2)	25.2 ± 2.6	25.1 ± 2.7	.965
Symptom duration (months)	25.3 ± 18.7	16.3 ± 11.5	.173
Previous treatment duration (months)	20.1 ± 19.9	8.4 ± 2.9	.068
Insole usage time (hour/day)	4.8 ± 2.6	3.4 ± 3.0	.875
Time to feel improvement with insole (weeks)	5.6 ± 2.2	4.8 ± 2.8	.630

TSI, three spike insole; TCI, total contact insole; BMI, body mass index

Non-inferiority analysis

Mean differences of VAS with 95% confidential interval (CI) at each time point of 6 weeks, 3 months, and 6 months were 0.6 (95% CI 0.6 – 2.6), 0.8 (95% CI -1.7 – 0.9), and 0.4 (95% CI -1.6 – 0.6), respectively. All value differences except for that of 6 weeks were within MID. The scale at 6 weeks showed superiority of TSI. These indicate that the TSI shows non-inferiority to TCI according to VAS.

Clinical outcomes

Almost all parameters except for KP score, Sport/Rec subscale in FAOS, and PCS in SF-36 showed significant improvement at 6 weeks in all participants ($p < .05$). Comparison between the groups are shown in Table 2 and Fig 3. All clinical outcome scores showed significant improvements over time in both groups on the basis of Friedman test ($p < .05$). Post hoc analysis with Mann-Whitney test conducted with Bonferroni correction further revealed that there were no significant differences of variables between the groups at baseline and 6 weeks; but many scales showed significant superiority of TSI at 3 months notably ($p < .0125$). Furthermore, KP score at 6 months was superior in TSI group ($p < .001$). We can conclude that the TSI group showed significantly superior results in all clinical outcomes to TCI group over time during 6 months of follow up.

Fig 3. Clinical outcomes of visual analog scale (VAS), American Orthopaedic Foot and Ankle Society (AOFAS) score, Karlsson-Peterson (KP) score, and Foot Function Index (FFI). TSI, three spike insole; TCI, total contact insole. * Significant p value < .05 between the groups. ├─┤ indicates overall significant improvement of the whole participants from the baseline.

Table 2. Comparisons of clinical outcomes between newly designed TSI and TCI groups.

	Baseline		6 weeks		3 months		6 months		Friedman test	
	TSI	TCI	TSI	TCI	TSI	TCI	TSI	TCI	Chi square	P value
VAS	6.2(1.5)	6.3(1.5)	3.4(2.1)	4.8(2.3)	3.4(1.7)	3.1(1.5)	2.4(1.3)	1.9(1.5)	61.1	**.001**
AOFAS score	59.2(13.0)	56.6(19.3)	77.5(10.1)	77.5(6.8)	77.0(11.2)	63.5(11.1)	75.4(11.6)	77.3(8.4)	23.7	**.001**
KP score	47.1(22.3)	57.1(25.9)	69.0(22.7)	66.1(17.4)	**76.1(13.1)**	**59.5(6.2)**	**91.9(4.5)**	**77.8(10.2)**	25.0	**.001**
FFI	85.3(30.3)	89.4(35.6)	70.6(39.9)	64.0(21.8)	**49.3(30.8)**	**79.4(17.6)**	44.0(37.7)	54.5(31.2)	33.6	**.001**
FAOS subscales										
Pain	38.7(18.3)	51.5(25.1)	63.5(21.6)	72.1(11.6)	**80.6(13.0)**	**54.9(7.6)**	74.0(5.8)	83.7(4.1)	25.7	**.001**
Symptoms	78.2(8.4)	76.6(7.3)	86.1(9.0)	85.0(5.9)	**87.7(6.4)**	**70.7(4.8)**	89.3(4.6)	86.5(4.3)	20.6	**.001**
ADL	74.8(14.4)	83.0(14.7)	87.0(10.5)	86.7(8.2)	88.7(7.9)	87.7(4.5)	85.5(4.5)	87.0(3.9)	10.6	**.014**
Sport/Rec	57.9(13.2)	69.6(28.5)	68.3(16.9)	78.0(8.8)	69.3(14.1)	73.4(5.2)	65.2(7.7)	67.7(11.6)	8.8	**.032**
QOL	24.0(18.4)	28.6(16.5)	41.7(24.5)	53.6(14.9)	43.8(31.9)	19.9(10.0)	57.8(21.1)	59.4(18.6)	32.7	**.001**
SF-36 subscales										
PCS	54.1(18.1)	56.8(15.8)	60.9(18.7)	63.3(12.9)	**63.3(16.8)**	**40.8(11.4)**	64.5(8.8)	71.8(8.1)	17.0	**.001**
MCS	64.3(18.0)	61.5(9.2)	78.0(12.7)	68.6(12.8)	**78.5(8.8)**	**53.1(7.0)**	79.6(4.2)	75.6(3.5)	20.5	**.001**

Boldface indicates statistical significance between the groups by post hoc analysis using Mann-Whitney test conducted with a Bonferroni correction ($p < .0125$). TSI, three spike insole; TCI, total contact insole; VAS, visual analog scale; AOFAS, American Orthopaedic Foot and Ankle Society; KP, Karlsson-Peterson; FFI, foot function index; FAOS, foot and ankle outcome score; ADL, activities of daily living; Sport/Rec, sport and recreational function; QOL, quality of life; SF-36, medical outcomes study short form-36; PCS, physical component summary; MCS, mental component summary

Discussion

This study showed that a newly designed TSI showed non-inferiority to conventional TCI according to VAS and a relatively rapid functional improvement during 6 month follow up. Both types of insoles were effective enough to reduce pain and increase pedal function and foot-related quality of life from mean 5.2 weeks of put on. Pain scale did not show significant differences at every time point between the groups and value differences of VAS were within MID. Moreover, KP score, FFI, pain and symptom subscales in FAOS, and SF-36 scores at 3 month were significantly superior in TSI group compared to TCI group. The KP score at 6 month in TSI group was significantly higher as well. These results imply that total contact support of medial longitudinal arch is not essential for symptom relief in plantar fasciitis. Previous biomechanical studies mentioned the requisitions of a proper insole for plantar fasciitis. Kogler et al. suggested that orthosis must lower the strain of the foot and the medial surface of the orthosis must stabilize the apical bony structure of the medial longitudinal arch to support it effectively.[22,25] Hsu et al. showed that the plantar fascial stress and peak pressure can be lowered by 14% and 38.9% with optimal insole that supports 54% contact surface of the medial longitudinal arch using finite element method.[21] Three spikes designed in this study were supposed to support more than 50% contact surface of the medial arch.

We can explain why sham orthoses have shown inferior results to custom or prefabricated orthoses with the above-mentioned reasons. Sham orthoses lack sufficient supportive structure for medial longitudinal arch. Landorf et al. compared sham orthosis, prefabricated orthosis, and customized orthosis in their randomized trial.[14] Pain and function scores favored prefabricated and customized orthosis over sham orthosis after 3 months. Bishop et al. compared custom orthosis with new shoes, sham insole with new shoes, and sham insole with patient’s regular shoes in a pragmatic randomized controlled study.[15] They concluded that custom orthosis with new shoes was superior in reducing pain and plantar fascia thickness over 12 weeks compared to new shoe alone or sham insole. Similarly, Oliveira et al. showed superior results of custom insole to flat insole with a 6 month follow up.[17] Pain scale and 6-min walk test were

significantly better in custom insole group. Bonanno et al. randomized 306 participants from ~~navy school~~ to a flat insole group and prefabricated orthosis group in their prospective study during a 11-week training.[26] Prefabricated orthosis group showed a 34% reduced risk of plantar fasciitis or Achilles tendinopathy. Both groups in the present study showed significant clinical improvements. Semi-rigidity and enough contact surface of the orthosis would have been the sufficient conditions to have a positive effect on plantar fasciitis ~~whatever the design is.~~

On the other hand, there have been many debates whether prefabricated orthosis is comparable with custom orthosis. Wrobel et al. compared custom foot orthoses, prefabricated foot orthoses, and sham insole on 77 patients in their randomized control study.[19] There were no significant differences among the groups for pain, FFI, and SF-36 for 3 months but the custom orthosis group demonstrated great improvements in spontaneous physical activity compared to prefabricated orthosis and sham insole group. Xu et al. randomized their 60 patients to customized 3D-printed orthoses and prefabricated orthoses groups.[20] The 3D-printed orthoses group showed superiority in comfort score even though the peak pressure in the hallux and first metatarsal area was significantly higher at initial. ~~While~~, Landorf concluded that there were no significant pain and functional improvement between prefabricated insole and custom insole.[14] Baldassin et al. compared the effectiveness of prefabricated and customized foot orthoses.[13] Both groups showed significant pain improvements from baseline to 4- and 8-week but there was no difference between the groups. Instead, they concluded low cost prefabricated orthosis made up of ethylene vinyl acetate would be the best choice for plantar fasciitis. These debatable results from many previous studies might vary from the shape of prefabricated insole used and subsequent arch support area that could be different in each participant. Provided the medial longitudinal arch of a participant was not sufficiently supported for over 50% surface of arch, the plantar fascial stress and peak pressure would not have been released enough to make obvious clinical results.

Therefore, the clinical results of this study are quite interesting. Newly designed TSI showed not only a

non-inferiority but also a significantly exceptional functional improvement and foot-related quality of life during a 6 month follow up. We postulate that these results came from the differences of bending moments in each model. When we assume the plantar fascia as a bow string, TCI can evenly support the fascial longitudinal arch with a uniformly distributed load. On [illegible]his wise, bending moments added on plantar fascia at some point in the model can be shown as Fig 4A.[23] Otherwise, TSI can support and stimulate the longitudinal arch at the specified 3 points as shown in Fig 4B. Bending moments at each point in this several concentrated load model tend to be bigger than those of the uniformly distributed load model. These bigger bending moments in TSI would have added more pressure on plantar fascia and would have led to consequent functional improvements. This multiple concentrated load distribution is easy to position according to the length of the patient's plantar fascia, and mass production of therapeutic insoles would be possible if three spikes are placed along the medial plantar arch of the insole for each size of ready made shoes.

Fig 4. A) When we assume that the plantar fascia is simply attached at the ends and the load (W, person's weight) is uniformly distributed, the bending moment at some point (s) can be calculated as formula M(s). Bending moments at each point of B, C, and D would be 0.8dW, 1.25dW, and 0.8dW if we assume that the point B and D are 2d away from each end. B) If multiple three concentrated loads are weighed on, the bending moments at each point of B, C, and D would be 1.0dW, 1.45W, and 1.0dW, respectively. R_A, reaction force at point A; R_E, reaction force at point E (each R_A and R_E can be calculated as '0.5W' here).

Our study has some limitations. First, the sample size is quite small to draw further differences or identities between the groups in this non-inferiority study model. However, similar demographic distribution to both groups and blinded physician who interpreted the patients' outcomes could have offset the possible bias. Although there was a 14.3% loss to follow-up over the course, it was equally allocated between the groups; and the remainders were followed up for a long period of 6 months. Further KP score, FFI, FAOS, and SF-36 used in this study are validated scores that effectively represent the physical activities and quality of life

of the patients. Second, lack of biomechanical study or a reliable plantar pressure measuring system could attenuate our explanation of functional superiority in TSI group. However, different bending moments between the groups according to the above-mentioned structural analysis could ~~explain the reason~~. Further biomechanical study of this design is needed to support the results of this investigation. Third, 3 persons in 24 participants (12.5%) dropped out because of the inconvenience of the 3D printed insole. The insole was somewhat inevitably rough and bulky to fit inside the patient's own shoes that could have led them to give up. An intention to treat analysis was performed but this could have lessened the strength of this study.

Conclusion

The present study assessed the effectiveness of a newly designed TSI compared to TCI in refractory plantar fasciitis. We reaffirmed that semi-rigid insole is effective in plantar fasciitis and showed a non-inferiority of TSI based on VAS. In addition, TSI more rapidly restored the pedal function compared to TCI. We assume that high bending moments in TSI model would have added more power on plantar fascia and have led consequent functional improvements. This newly designed TSI model can be not only effective in deriving better clinical outcomes but also be manufactured for popularization due to its reproducible design.

REFERENCES

1. Crawford F, Thomson C. Interventions for treating plantar heel pain. Cochrane Database Syst Rev. 2003: CD000416. doi:10.1002/14651858.CD000416 PMID:12917892
2. Gill LH. Plantar Fasciitis: Diagnosis and Conservative Management. J Am Acad Orthop Surg. 1997;5: 109-117. doi:10.5435/00124635-199703000-00006 PMID:10797213
3. Ogden JA, Alvarez RG, Levitt RL, Johnson JE, Marlow ME. Electrohydraulic high-energy shock-wave treatment for chronic plantar fasciitis. J Bone Joint Surg Am. 2004;86: 2216-2228. doi:10.2106/00004623-200410000-00013 PMID:15466731
4. Rompe JD, Schoellner C, Nafe B. Evaluation of low-energy extracorporeal shock-wave application for treatment of chronic plantar fasciitis. J Bone Joint Surg Am. 2002;84: 335-341. doi:10.2106/00004623-200203000-00001 PMID:11886900
5. DiGiovanni BF, Nawoczenski DA, Lintal ME, Moore EA, Murray JC, Wilding GE, et al. Tissue-specific plantar fascia-stretching exercise enhances outcomes in patients with chronic heel pain. A prospective, randomized study. J Bone Joint Surg Am. 2003;85: 1270-1277. doi:10.2106/00004623-200307000-00013 PMID:12851352
6. Roos E, Engstrom M, Soderberg B. Foot orthoses for the treatment of plantar fasciitis. Foot Ankle Int. 2006;27: 606-611. doi:10.1177/107110070602700807 PMID:16919213
7. Yucel U, Kucuksen S, Cingoz HT, Anliacik E, Ozbek O, Salli A, et al. Full-length silicone insoles versus ultrasound-guided corticosteroid injection in the management of plantar fasciitis: a randomized clinical trial. Prosthet Orthot Int. 2013;37: 471-476. doi:10.1177/0309364613478328 PMID:23471226
8. Baxter DE, Thigpen CM. Heel pain--operative results. Foot Ankle. 1984;5: 16-25. doi:10.1177/107110078400500103 PMID:6479759
9. Davies MS, Weiss GA, Saxby TS. Plantar fasciitis: how successful is surgical intervention? Foot Ankle Int. 1999;20: 803-807. doi:10.1177/107110079902001209 PMID:10609710
10. Conflitti JM, Tarquinio TA. Operative outcome of partial plantar fasciectomy and neurolysis to the nerve of the abductor digiti minimi muscle for recalcitrant plantar fasciitis. Foot Ankle Int. 2004;25: 482-487. doi:10.1177/107110070402500707 PMID:15319106
11. Sammarco GJ, Helfrey RB. Surgical treatment of recalcitrant plantar fasciitis. Foot Ankle Int. 1996;17: 520-526. doi:10.1177/107110079601700902 PMID:8886777

12. Riddle DL, Pulisic M, Pidcoe P, Johnson RE. Risk factors for Plantar fasciitis: a matched case-control study. J Bone Joint Surg Am. 2003;85: 872-877. doi:10.2106/00004623-200305000-00015 PMID:12728038
13. Baldassin V, Gomes CR, Beraldo PS. Effectiveness of prefabricated and customized foot orthoses made from low-cost foam for noncomplicated plantar fasciitis: a randomized controlled trial. Arch Phys Med Rehabil. 2009;90: 701-706. doi:10.1016/j.apmr.2008.11.002 PMID:19345789
14. Landorf KB, Keenan AM, Herbert RD. Effectiveness of foot orthoses to treat plantar fasciitis: a randomized trial. Arch Intern Med. 2006;166: 1305-1310. doi:10.1001/archinte.166.12.1305 PMID:16801514
15. Bishop C, Thewlis D, Hillier S. Custom foot orthoses improve first-step pain in individuals with unilateral plantar fasciopathy: a pragmatic randomised controlled trial. BMC Musculoskelet Disord. 2018;19: 222. doi:10.1186/s12891-018-2131-6 PMID:30021556
16. Gross MT, Byers JM, Krafft JL, Lackey EJ, Melton KM. The impact of custom semirigid foot orthotics on pain and disability for individuals with plantar fasciitis. J Orthop Sports Phys Ther. 2002;32: 149-157. doi:10.2519/jospt.2002.32.4.149 PMID:11949663
17. Oliveira HA, Jones A, Moreira E, Jennings F, Natour J. Effectiveness of total contact insoles in patients with plantar fasciitis. J Rheumatol. 2015;42: 870-878. doi:10.3899/jrheum.140429 PMID:25774062
18. Walther M, Kratschmer B, Verschl J, Volkering C, Altenberger S, Kriegelstein S, et al. Effect of different orthotic concepts as first line treatment of plantar fasciitis. Foot Ankle Surg. 2013;19: 103-107. doi:10.1016/j.fas.2012.12.008 PMID:23548451
19. Wrobel JS, Fleischer AE, Crews RT, Jarrett B, Najafi B. A randomized controlled trial of custom foot orthoses for the treatment of plantar heel pain. J Am Podiatr Med Assoc. 2015;105: 281-294. doi:10.7547/13-122.1 PMID:25941995
20. Xu R, Wang Z, Ma T, Ren Z, Jin H. Effect of 3D Printing Individualized Ankle-Foot Orthosis on Plantar Biomechanics and Pain in Patients with Plantar Fasciitis: A Randomized Controlled Trial. Med Sci Monit. 2019;25: 1392-1400. doi:10.12659/msm.915045 PMID:30789873
21. Hsu YC, Gung YW, Shih SL, Feng CK, Wei SH, Yu CH, et al. Using an optimization approach to design an insole for lowering plantar fascia stress--a finite element study.

Ann Biomed Eng. 2008;36: 1345-1352. doi:10.1007/s10439-008-9516-x PMID:18481179

22. Kogler GF, Solomonidis SE, Paul JP. Biomechanics of longitudinal arch support mechanisms in foot orthoses and their effect on plantar aponeurosis strain. Clin Biomech (Bristol, Avon). 1996;11: 243-252. doi:10.1016/0268-0033(96)00019-8 PMID:11415628

23. Hibbeler RC, Kiang T. Structural analysis: Pearson Prentice Hall; 2015.

24. Landorf KB, Radford JA, Hudson S. Minimal Important Difference (MID) of two commonly used outcome measures for foot problems. J Foot Ankle Res. 2010;3: 7. doi:10.1186/1757-1146-3-7 PMID:20465855

25. Kogler GF, Solomonidis SE, Paul JP. In vitro method for quantifying the effectiveness of the longitudinal arch support mechanism of a foot orthosis. Clin Biomech (Bristol, Avon). 1995;10: 245-252. doi:10.1016/0268-0033(95)99802-9 PMID:11415561

26. Bonanno DR, Murley GS, Munteanu SE, Landorf KB, Menz HB. Effectiveness of foot orthoses for the prevention of lower limb overuse injuries in naval recruits: a randomised controlled trial. Br J Sports Med. 2018;52: 298-302. doi:10.1136/bjsports-2017-098273 PMID:29056595

Figure 1

Click here to access/download;Figure;Figure 1.jpg

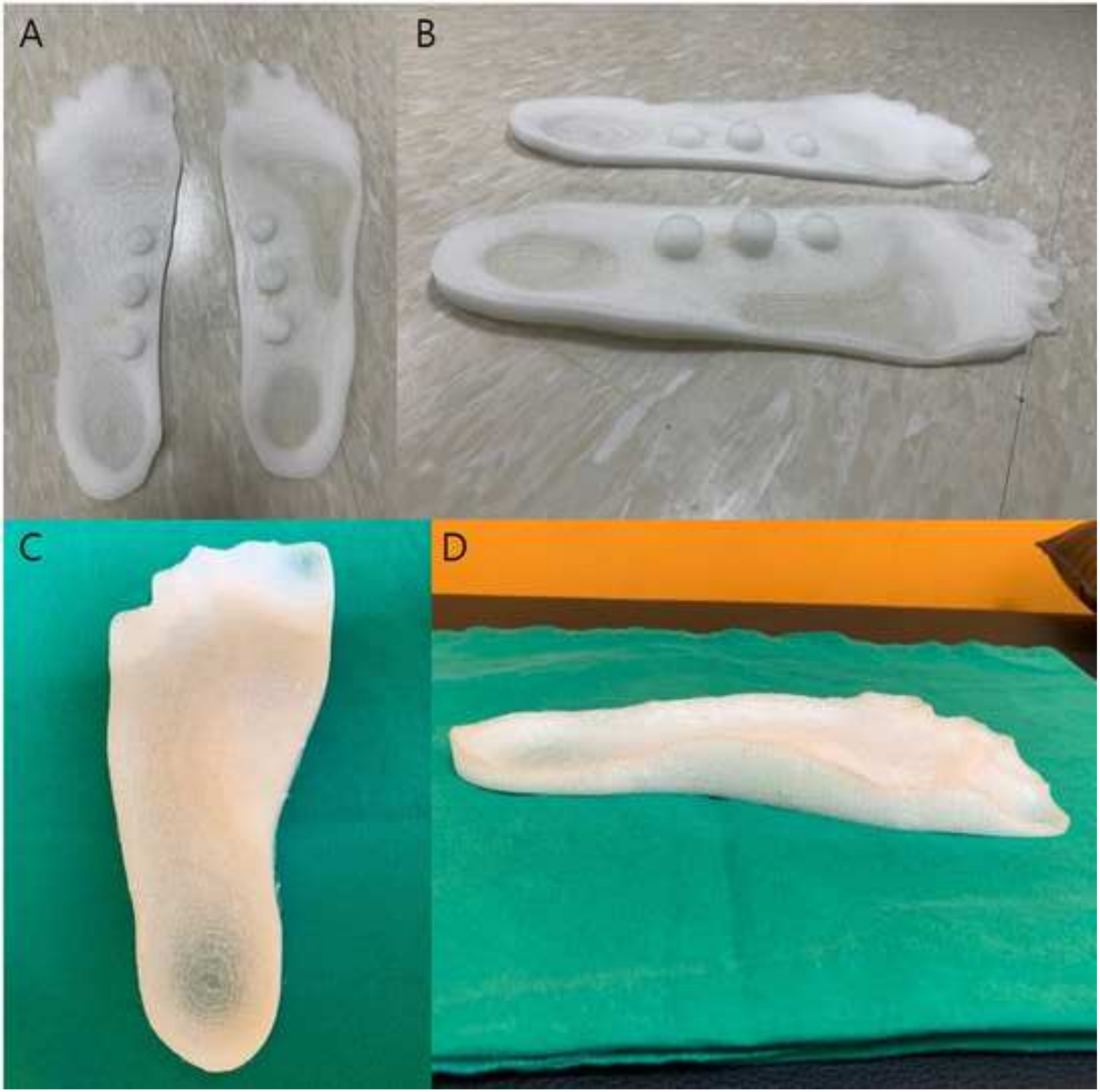

Click here to access/download;Figure;Figure 2.jpg

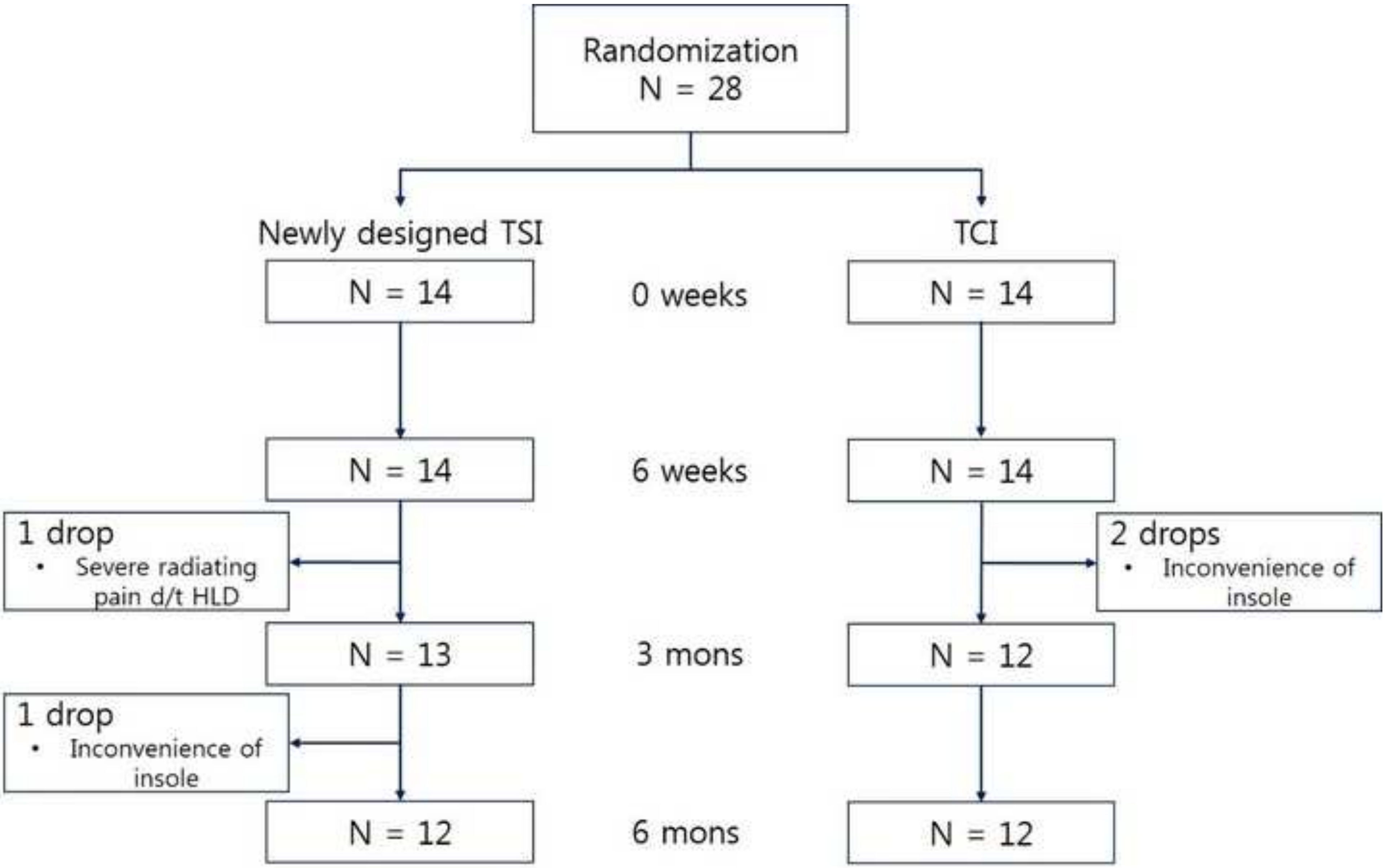

Figure 3

Click here to access/download;Figure;Figure 3.jpg

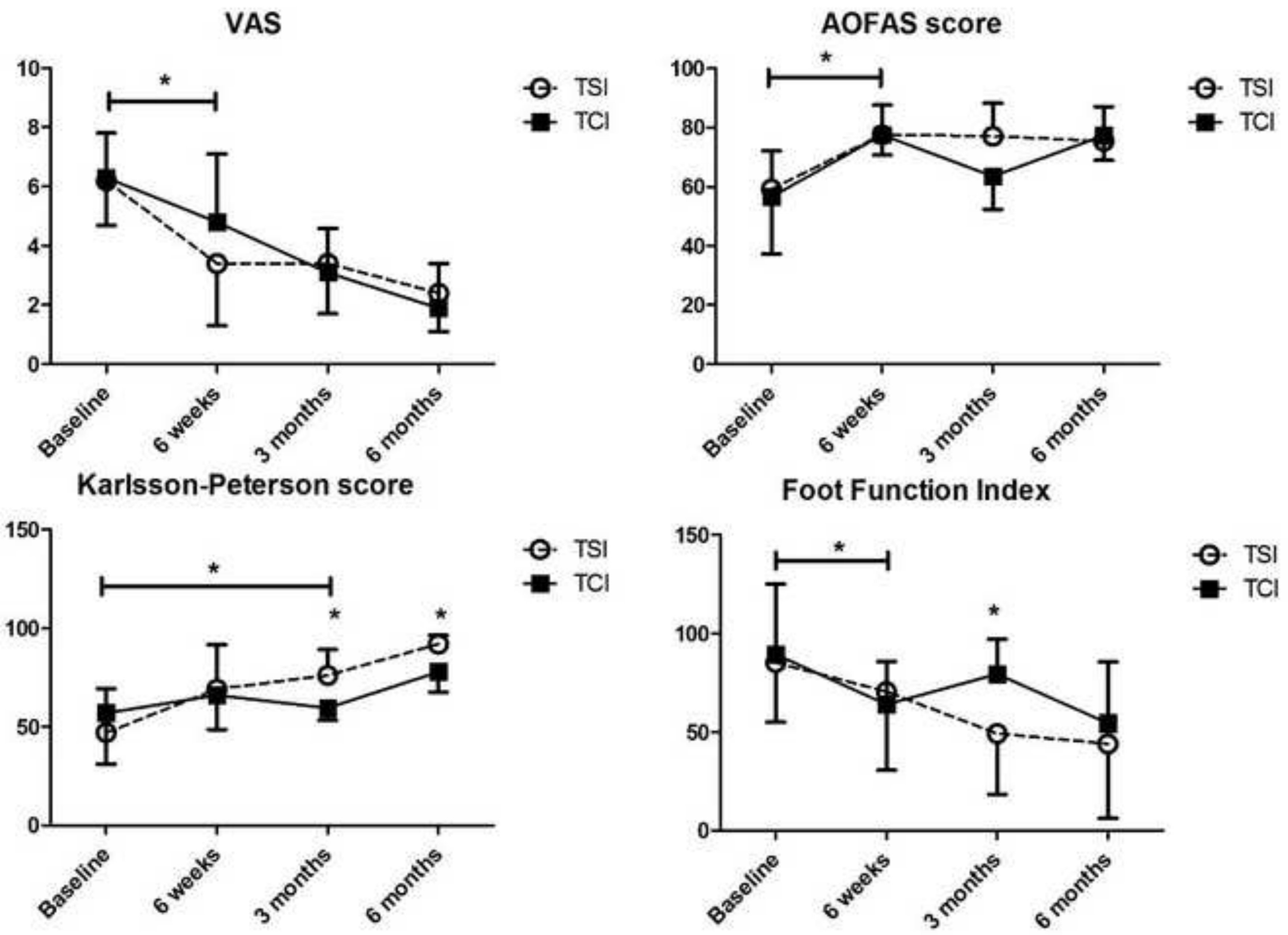

Figure 4

Click here to access/download;Figure;Figure 4.jpg

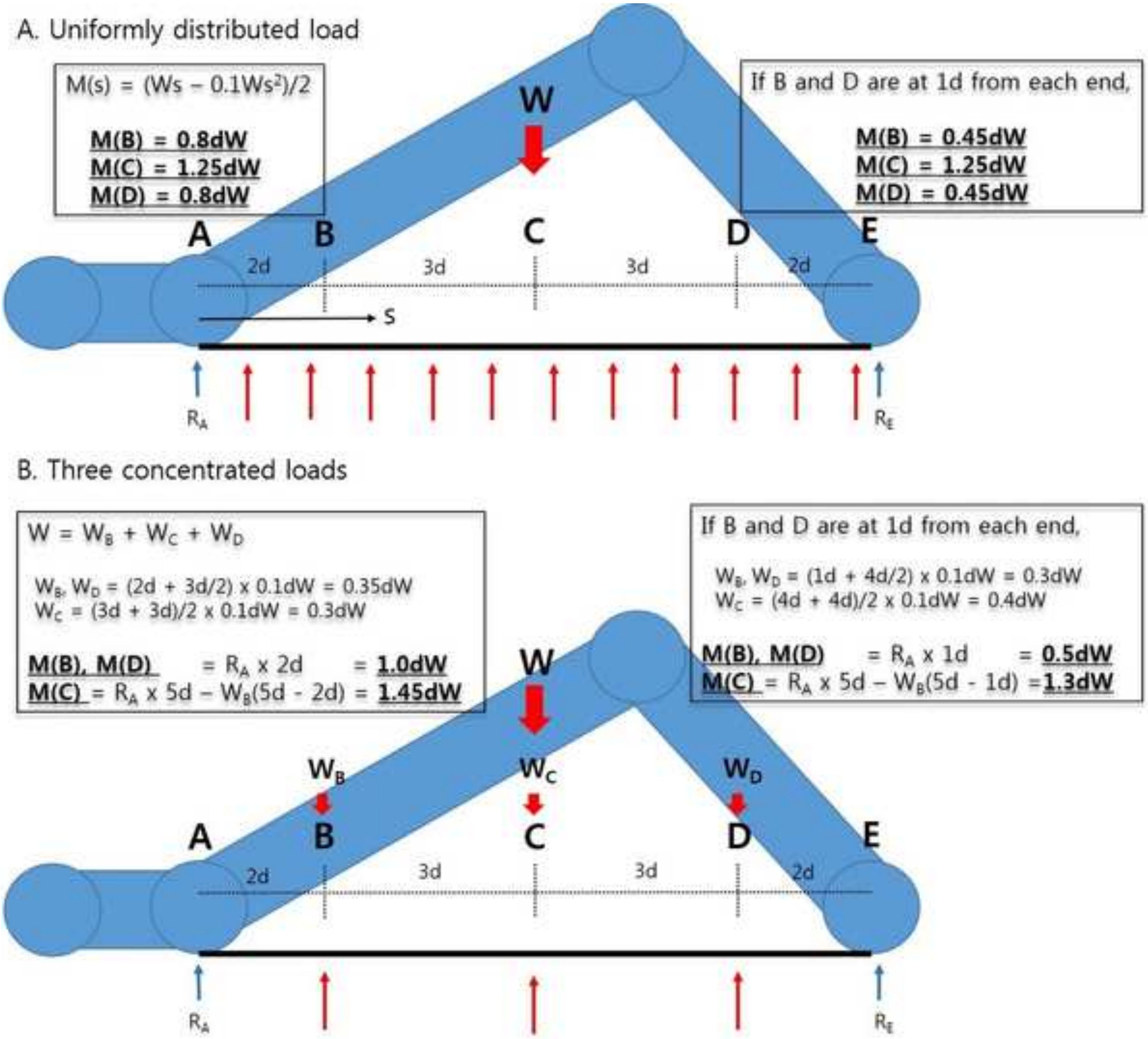

Consort checklist

Click here to access/download
Supporting Information
CONSORT 2010 Checklist.doc

Click here to access/download
Supporting Information
8-1. (translation)Study protocol.doc

Study protocol(original)

Click here to access/download
Supporting Information
(original language) study protocol.doc

IRB approval

Click here to access/download
Supporting Information
renamed_4169b.pdf

IRB approval (Original language)

Click here to access/download
Supporting Information
renamed_ffadc.pdf
