# Supplementary material for: Superior pedal function recovery of newly designed three spike insole over total contact insole in refractory plantar fasciitis: A randomized, double-blinded, non-inferiority study
Source: PLoS One. 2021 Jul 23;16(7):e0255064. doi: 10.1371/journal.pone.0255064 (PMC8301654; doi:10.1371/journal.pone.0255064)
Supplement: Attachment — Submitted filename: Response to reviewers.docx [file pone.0255064.s006.docx]

Please include the following items when submitting your revised manuscript:

- A rebuttal letter that responds to each point raised by the academic editor and reviewer(s). You should upload this letter as a separate file labeled 'Response to Reviewers'.
- A marked-up copy of your manuscript that highlights changes made to the original version. You should upload this as a separate file labeled 'Revised Manuscript with Track Changes'.
- An unmarked version of your revised paper without tracked changes. You should upload this as a separate file labeled 'Manuscript'.

When submitting your revision, we need you to address these additional requirements.

1. Please ensure that your manuscript meets PLOS ONE's style requirements, including those for file naming. The PLOS ONE style templates can be found at

<https://journals.plos.org/plosone/s/file?id=wjVg/PLOSOne_formatting_sample_main_body.pdf> and

[https://journals.plos.org/plosone/s/file?id=ba62/PLOSOne_formatting_sample_title_authors_affiliations.pdf](about:blank)

*→ Thank you for your pointing out. We reaffirmed that we have revised the format as introduced and met the requirements.*

2. Thank you for submitting your clinical trial to PLOS ONE and for providing the name of the registry and the registration number. The information in the registry entry suggests that your trial was registered after patient recruitment began. PLOS ONE strongly encourages authors to register all trials before recruiting the first participant in a study.

  As per the journal’s editorial policy, please include in the Methods section of your paper:

1) your reasons for your delay in registering this study (after enrolment of participants started);

*→ Thank you for your pertinent comment. We commented the reason of delay. Actually, we had no idea of the public registration at the beginning of this study. (Line 98-99)*

2) confirmation that all related trials are registered by stating: “The authors confirm that all ongoing and related trials for this drug/intervention are registered.

*→ Thank you for your comment. We added the sentence right after the above mentioned amendment. (Line 99-101)*

3. We suggest you thoroughly copyedit your manuscript for language usage, spelling, and grammar. If you do not know anyone who can help you do this, you may wish to consider employing a professional scientific editing service.

  Whilst you may use any professional scientific editing service of your choice, PLOS has partnered with both American Journal Experts (AJE) and Editage to provide discounted services to PLOS authors. Both organizations have experience helping authors meet PLOS guidelines and can provide language editing, translation, manuscript formatting, and figure formatting to ensure your manuscript meets our submission guidelines. To take advantage of our partnership with AJE, visit the AJE website (http://learn.aje.com/plos/) for a 15% discount off AJE services. To take advantage of our partnership with Editage, visit the Editage website (www.editage.com) and enter referral code PLOSEDIT for a 15% discount off Editage services.  If the PLOS editorial team finds any language issues in text that either AJE or Editage has edited, the service provider will re-edit the text for free.

 Upon resubmission, please provide the following:

●             The name of the colleague or the details of the professional service that edited your manuscript

●             A copy of your manuscript showing your changes by either highlighting them or using track changes (uploaded as a *supporting information* file)

●             A clean copy of the edited manuscript (uploaded as the new *manuscript* file)

*→ Thank you for your suggestion. We have made our manuscript edited once again via BIENTrans and uploaded the certificate as well.*

4. We note that you have indicated that data from this study are available upon request. PLOS only allows data to be available upon request if there are legal or ethical restrictions on sharing data publicly. For information on unacceptable data access restrictions, please see http://journals.plos.org/plosone/s/data-availability#loc-unacceptable-data-access-restrictions.

  In your revised cover letter, please address the following prompts:

 4a) If there are ethical or legal restrictions on sharing a de-identified data set, please explain them in detail (e.g., data contain potentially identifying or sensitive patient information) and who has imposed them (e.g., an ethics committee). Please also provide contact information for a data access committee, ethics committee, or other institutional body to which data requests may be sent.

 4b) If there are no restrictions, please upload the minimal anonymized data set necessary to replicate your study findings as either Supporting Information files or to a stable, public repository and provide us with the relevant URLs, DOIs, or accession numbers. Please see http://www.bmj.com/content/340/bmj.c181.long for guidelines on how to de-identify and prepare clinical data for publication. For a list of acceptable repositories, please see <http://journals.plos.org/plosone/s/data-availability#loc-recommended-repositories>.

 We will update your Data Availability statement on your behalf to reflect the information you provide.

*→ Thank you for your reminder. We uploaded our data set as Supporting Information and have mentioned it in the revised cover letter.*

5. Please include captions for your Supporting Information files at the end of your manuscript, and update any in-text citations to match accordingly. Please see our Supporting Information guidelines for more information: http://journals.plos.org/plosone/s/supporting-information.

*→ Thank you for your guidance. We have added a sentence at the end of the manuscript.*

 6. Please review your reference list to ensure that it is complete and correct. If you have cited papers that have been retracted, please include the rationale for doing so in the manuscript text, or remove these references and replace them with relevant current references. Any changes to the reference list should be mentioned in the rebuttal letter that accompanies your revised manuscript. If you need to cite a retracted article, indicate the article’s retracted status in the References list and also include a citation and full reference for the retraction notice.

*→ Thank you for your reminder. We have checked listed references once again and they are complete and correct.*

Additional Editor Comments:

Thanks for contributing your original study to PLOS One. It was reviewed by three independent reviewers with complementary expertise in podiatric medicine, biomechanics, and wearables. All reviewers agreed that this study has scientific merits but could be improved further and provided excellent comments. These comments should be addressed before I could recommend acceptance of your manuscript for publication in PLOS One.


[Note: HTML markup is below. Please do not edit.]

Reviewers' comments:

5. Review Comments to the Author

Please use the space provided to explain your answers to the questions above. You may also include additional comments for the author, including concerns about dual publication, research ethics, or publication ethics. (Please upload your review as an attachment if it exceeds 20,000 characters)

Reviewer #1: A randomized controlled noninferiorty clinical trial was conducted to compare VAS scores of the total contact sole (TCS) with a newly designed three-spike insole (TSI). The study results indicated that the experimental design was noninferior to the standard insole design. Improvements over time were noted in both groups.
Minor revisions:
1- Abstract: Rephrase to improve clarity: “Twenty-eight patients were randomized with equal allocation to either TSI or TCI.”

*→ We thank you for your clarification. We have amended it as you have guided.*

2- Express numerical p-values more precisely, rather than p < 0.0xx.

*→ We thank you for these very important comments. We have proposed more precise ones as you have commented. (Line 51, 53, 190, 192, 195)*

3- Line 112: Specify the block size used in the randomization.

*→ We thank you for these pertinent comments. We used four block randomization and added it as you have recommended. (Line 103)*

4- Line 162: Use standard phrasing: “Twenty-eight patients were randomized, with equal probability, to two groups.”

*→ We thank you for your clarification. We revised it as you have recommended. (Line 154)*

5- For normally distributed variables, summarize with mean and standard deviation. For nonparametric variables, summarize using median, first, and third quartiles.

*→ We thank you for this perceptive comment. We added standard deviation of each value right after mean value. (Line 158-160)*

6- Indicate if any adverse events occurred during the course of the study.

*→ Thank you for your comment. There was no adverse effect except for the dropped cases as we described in CONSORT flowchart. We specified the absence of adverse event (Line 197)*

Reviewer #2: The authors conducted a randomized controlled trial comparing the effect of newly designed insole and total contact insole on plantar fasciitis symptoms. Overall, this study seems well-conducted with reasonable designs and results. I have a few comments.
Introduction
In the first paragraph, please include some data from Korea, if available. For example, the prevalence of plantar fasciitis in Korea, economic cost, etc.

*→ Thank you for your comment. Unfortunately, there was no study on prevalence of plantar fasciitis in Korea. However, we added the information of the economic cost in Korea in accordance with National Health Insurance Service. (Line 63-65)*

The primary aim is to test new spike insole, and yet it is not very clear to me what are the problems or limitations with conventional insoles. Is the problem just the cost and the duration of fabrication? Also, why did the authors come up with the spike, that is why did the authors believe the spiky design would address the limitations of conventional insoles?
The purpose statement needs to be added.

*→ Thank you for your pertinent question. As you have mentioned again, the conventional insoles are quite expensive and take time for customized production. However, we focused on that partially arch supporting structured insole could have a comparable load distribution effect on plantar fascia. Moreover, we postulated that if it has stimulation effect on both ends of fascia, the fascia releasing effect could be maximized. That is why we initially designed the three-spike insole. This design has the merit over conventional TCI on that the three spikes can be easily located uniformly along the plantar fascia. It can be applied to the insoles commonly used in the market with the effect of fascial stretching, saving time and money compared to a TCI and thus allowing mass production of therapeutic insole with the above design. However, the purpose does not contain the exact content as you pointed. We revised it. (Line 82)*

Methods
In the figure 1, please add a side view photo so that readers can see the height of spikes.

*→ Thank you for your recommendation. We added the lateral view of each insole in Fig 1.*

Also, by any chance, did the authors have any data from patients' perceive satisfaction for the new insole? If so, please add the results.

*→ Thank you for your question. Unfortunately, we did not check the patient reported satisfaction scale for each insole.*

Why did the authors use Mann-Whitney? All the variables were non-parametric? Please confirm.

*→ We thank you for your perceptive comment. We used Mann-Whitney and Fisher’s exact test conservatively because many but not all values showed non-parametric distribution.*

Results
The figure 2 is difficult to understand. What exactly the asterisk represent? Significant difference in the spike insole group or total contact insole? Do the asterisks represent time x group effect or just time effect?

*→ We are grateful for you pointing out this ambiguity. We guess you are pointing Figure 3. Captions might have insufficient explanation. Individual asterisk points significant difference between the groups. And the bar indicates the overall improvement from baseline in both groups. We removed the asterisk over the bar since it was confusing.*

No new figure should be introduced in discussion so please move figure 4 to results section, and describe the results in this section.

*→ Thank you for your recommendation. We stated the calculation of bending moments at the end of the Material and Methods section (Line 127-128) and moved the figure and results to the Result section. (Line 169-180)*

Discussion and conclusion seems to be fine.

Reviewer #3: The authors present a well designed and written study evaluating the efficacy of a novel “3-spike” design shoe insert for the treatment of plantar fasciitis recalcitrant to prior treatment.
To evaluate efficacy the authors enrolled 14 patients to each arm of a non-inferiority comparison between standard orthotic device and novel interventional device. Patients were tracked for 6 months and evaluated using multiple validated subjective scoring systems.

The interventional inserts were created using molds of the patient’s feet, and were 3-D printed to match the patient's weightbearing medial arch.

Utilizing prior experience and observational information regarding the nature of the mold the length and position of the medial arch band of the plantar fascia was interpreted and insoles were either made to be A: load bearing along the full length (Control), or B: fitted with three 2cm diameter elevated “spikes” at center point and distal/proximal along the medial band.
Ultimately the authors found the patient's pain and functional capacity were normalized faster utilizing novel design as compared to standard of care design (control). In this regard the author’s conclude this novel technique to be at minimum non-inferior and likely superior to standard of care.

The authors chose to craft booth insoles using similar methodology to control for variations in design other than the interventional modification. The authors state their believed limitations of this study regarding limited treatment group size and lack of biomechanical or pressure data to support hypothesis of function and loading on novel device. Despite these limitations the authors have designed and executed a well-developed study with novel implications for clinical management of a frequently encountered condition.

I recommend this article for acceptance with minor grammatical revision and look forward to future work from these authors in this field.

*→ We are sincerely grateful for your compliments. We are preparing the subsequent large cohort study and hope to introduce it to you in the future.*
